# LATE SURGICAL TREATMENT FOR SPONTANEOUS RUPTURE OF HEPATOCELLULAR ADENOMA: CASE REPORT

**DOI:** 10.1590/S0102-6720201500030022

**Published:** 2015

**Authors:** Luis Eduardo Veras PINTO, João Paulo Ribeiro SILVA, Gustavo Coêlho RÊGO, José Huygens Parente GARCIA

**Affiliations:** 1 Department of Surgery, University Hospital Walter Cantídio, Digestive Surgery Service, Federal University of Ceará, Fortaleza, CE, Brazil

## INTRODUCTION

Hepatocellular adenoma (HA) is a rare benign neoplasm of the liver. It is strongly associated with oral contraceptive used by woman in childbearing age, by men receiving anabolic steroids and metabolic diseases. HA incidence has raised over the years from 5 per 1 milion in 1964^9^ to 4 per 100.000^3^ , for unknown reasons.

 HA is important because of its risk of complications such as life threatening rupture of the tumor and malignant transformation. Surgical treatment in ruptured HA has a high mortality rate but it is still the standard procedure in those cases^5^. Emergency liver resection in ruptured HA has a mortality rate from 5-10%, but in elective resections the mortality rate is under 1%.

Different procedures have been suggested to decrease mortality rates and spare liver parenchyma, such as arterial embolization of ruptured adenomas, although it is not an available procedure in most centers.

## CASE REPORT

A 44-year-old woman admitted in the emergency department of another hospital with complaints of sudden upper quadrant abdominal pain. She had been using oral contraceptives for 31 years. She presented with acute mild abdominal pain in epigastrium as well as right hypochondrium pain followed by light dyspnea and dizziness. She was treated with analgesics and ordered an abdominal ultrasound made only three days after the pain. The ultrasound showed a solid liver mass in the right hepatic lobe measuring 150x100x100 mm^3.^ She was discharged without additional treatment.

After almost five months after the occurrence, she was referred to our surgical department for diagnostic investigation after another episode of abdominal pain just like the last time, followed by tachycardia and cold sweating. Physical examination revealed mild anemia and a diffuse abdominal pain, without peritonitis or palpable masses.

Lab exams showed 8.61g/dl haemoglobin, white blood count 8.980/mm^3^ and 214.00 platelets. Liver transaminases level were altered: ALT 306 IU/l, AST 154 IU/l, alkaline phosphatase 142 IU/l and gamma-glutamyl transferase 229 IU/l. Alfa-fetoprotein and clotting functions were normal.

**FIGURE 1:**
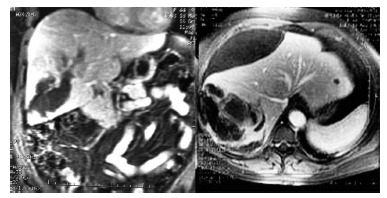
- MRI showing adenoma and bulky subcapsular hematoma

Abdominal CT and MRI showed an enlarged liver, with a contrast enhanced liver mass involving segments VI and VII. The liver mass measured 118x70mm^2^ associated with a perilesional voluminous hematoma measuring 8cm and haemoperitoneum. The radiologic findings suggested a ruputured hepatocellular adenoma (Figure 1)

**FIGURE 2:**
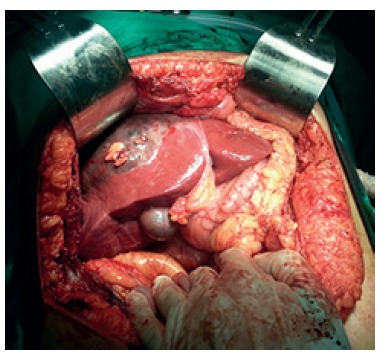
- Liver adenoma and subcapsular hematoma envolving all right hepatic lobe

**FIGURE 3:**
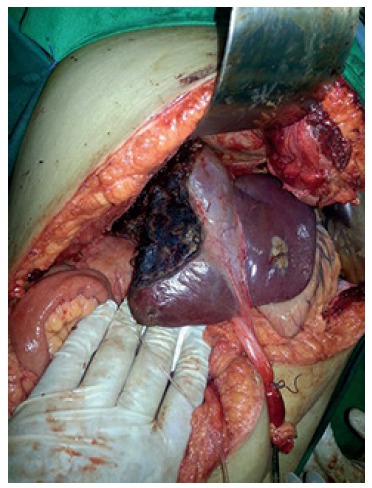
- Liver remnant after anatomic right hepatectomy (round ligament pulled caudally)

Patient was admitted and sent to observation room to be prepared for surgical procedure after clinical improvement. She was transfused with two bags of packed red bloods cells and intensive electrolytes control. Patient was sent to the operation room and had an open laparotomy with Chevron incision. During operative exploration was found a voluminous liver lesion involving segments VI and VII, large subcapsular hematoma over the right hepatic lobe and mild hemoperitoneum. The right lobe of the liver had strong adhesions to the right diaphragm. It was decided to make an anterior approach with inflow control due to the risk of bleeding, by ligation of the right portal vein and the right hepatic artery. The procedure continued with parenchyma right transection in the cantlie´s line, showed by isquemic demarcation, using bipolar forceps, argon coagulator device and kelly-crush technique. The liver parenchyma was dissected by the hematoma in some parts, disarranging the liver architecture. In the last part of the procedure, was mobilized all the right liver lobe followed by outflow control, by ligation of the right hepatic vein. During the liver mobilization, was able to see a large rupture in the posterior part of the liver and multiple adhesions between liver parenchyma and diaphragm, but without any sign of active bleeding. The surgery was completed with an anatomic right hepatectomy (Figures 2 and 3)

The surgical specimen weighted 1.170 g, measured 18,0x14,0x12,0 cm, presenting with a 12 cm rupture, externalizing an irregular and hemorrhagic mass. The tumor measured 1,0x6,0x6,0 cm, friable, along with a large subcapsular hematoma and clear margins.

**FIGURE 4:**
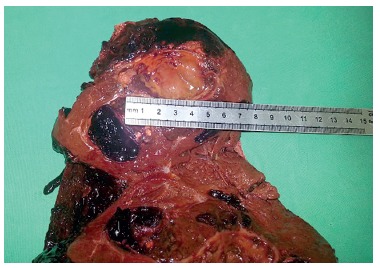
- Hepatic adenoma of 6 cm

**FIGURE 5:**
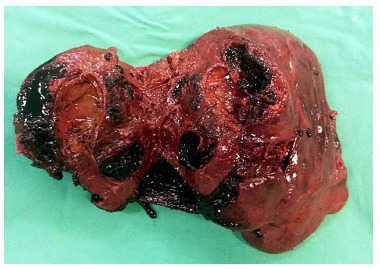
- Right hepatic lobe oppened with the presence of adenoma and multiple clots infiltrating hepatic parenchyma

Microscopy showed a neoplasm filled with hepatocytes cells showing enlarged plasmatic volume, eosinophils, macrovesicular steatosis, regular nucleus with minimum atypia and rare nucleolus. There was sinusoidal dilatation with spots of necrosis and hemorrhage. All these data corroborate with the hepatocellular adenoma (Figures 4 and 5).

Patient was kept in the intensive care unit for a day. There were no blood transfusions and the patient was discharged in the 6^th^ operative day without any complications.

## DISCUSSION

HA is a rare condition, and is commonly associated with oral contraceptive use^6^. The longer women uses oral contraceptives with increased estrogen level, the higher is the chance to develop hepatocellular adenomas. It´s frequently seen in woman in childbearing age, as well as in men and diseases like hemochromatosis and type 1 glycogen storage disease^4^. It has clinical importance because of the risk of complications. Spontaneous rupture is the most important complication and usually happens in adenomas greater than 5 cm, in 20-40% of the cases^4^.

Approximately 10% of patients with HA present with acute abdominal pain due to rupture and hemoperitoneum, in some cases followed by hipovolemic shock. Patients might also refer nauseas, vomiting, anorexia and fever. Mortality in ruptured HA has been associated with late diagnosis, coagulopathy and post-operative complications^8^.

Conservative treatment is used to small adenomas, mainly the ones related to oral contraceptives and anabolic steroids. The follow up in these cases includes abdominal TC or ultrasound each sux months. Although, when the adenoma is higher than 5cm or show symptoms, the surgical treatment is recommended because of the increased risk of hemorrhage and malignant transformation^6^.

The surgical treatment vary on the case, as well as the different approaches of liver resection. The standard treatment for ruptured HA must be local or segmental resections, to spare as much liver parenchyma as possible. Laparoscopic hepatectomy is a feasible option for benign liver lesions in elective cases^1^^,^ 2, but when it comes to ruptured, the open laparotomy is preferred due to bleeding and close contact to important liver vessels. Patients with ruptured HA must have elective resections when possible. There are several procedures to avoid emergency treatment but surgery still remains the main approach. If patient is stable and the tumor is localized in one segment, partial hepatectomy should be promptly made, but if there is important blood loss, volemic reposition with fluids and transfusions of hemocomponents should be attempted as well as arterial embolization^10^, with late resection when patient is stable. The selective arterial embolization have been playing an important role in the treatment of ruptured and non-ruptured HA, with low complication rates when compared with emergency surgery^7^. Arterial embolization is a very expensive procedure and is not available in most centers.
